# The Effects of a Custom−Designed High−Collar Shoe on Muscular Activity, Dynamic Stability, and Leg Stiffness: A Biomimetic Approach Study

**DOI:** 10.3390/biomimetics8030274

**Published:** 2023-06-27

**Authors:** Alireza Nasirzadeh, Jaeha Yang, Seungtae Yang, Juseok Yun, Young Yoon Bae, Juyeon Park, Jooeun Ahn, Giuk Lee

**Affiliations:** 1 Department of Mechanical Engineering, Chung-Ang University, Seoul 06974, Republic of Korea; alireza@cau.ac.kr (A.N.);; 2 HUROTICS Inc., Seoul 06974, Republic of Korea; 3 Department of Fashion and Textiles, Seoul National University, Seoul 08826, Republic of Korea; 4 Department of Physical Education, Seoul National University, Seoul 08826, Republic of Korea; 5 Institute of Sport Science, Seoul National University, Seoul 08826, Republic of Korea

**Keywords:** ankle sprain prevention, high-collar shoes, collar stiffness, circumferential ankle pressure, running biomechanics

## Abstract

High-collar shoes are a biomimetic approach to preventing lateral ankle injuries during high-demand activities; however, the influence of collar stiffness (CS) on parameters related to lateral ankle sprain prevention during running remains unclear. In this study, we investigated the effects of a custom-designed shoe CS on muscular activity, dynamic stability, and leg stiffness (K_leg_) during running using a biomimetic design approach inspired by the mechanisms of ankle sprain prevention. Sixteen healthy male participants ran on a treadmill while wearing a custom-designed high-collar shoe with low, medium, and high CS conditions, measured using circumferential ankle pressure (CAP). Lower extremity kinematics and electromyography (EMG) data were recorded simultaneously. One-way repeated-measures ANOVA was conducted to compare the CS conditions. Results indicate that high and medium CS conditions significantly reduce sagittal and frontal ankle ranges of motion (ROMs) compared to the low CS condition, providing improved stability and support against lateral ankle sprain; moreover, there was a trend towards higher dynamic stability and K_leg_ with increasing CS. Our study highlights the importance of considering the CAP in regulating high-collar stiffness properties and how higher CS may provide better support for the ankle during running. Nevertheless, additional research is necessary to validate the efficacy of the current design in preventing ankle sprains during high-demand activities.

## 1. Introduction

Ankle injuries are a common occurrence in sports and physical activity and often result from sudden twisting or rolling of the ankle joint, leading to sprains or ligamentous tears [1]. The ankle joint’s stability is mainly provided by the ligaments, muscles, and bones surrounding it; however, these structures may not always be sufficient to prevent injury, particularly during dynamic movements such as running or jumping. Therefore, a biomimetic approach could be taken to design high-collar shoes that mimic the ankle’s natural stability mechanisms to prevent injuries. A high-collar shoe can provide external support and stability to the ankle joint, mimicking the function of the ankle ligaments. Moreover, a high-collar shoe can be designed with adjustable collar stiffness, mimicking the activation of ankle muscles to provide dynamic stability during movement [2]. The combination of these biomimetic design features can help reduce the risk of ankle injuries during physical activity, particularly in athletes who are at a higher risk of ankle sprains.

To protect the ankle in the lateral direction and improve stability, the shoe’s collar should provide sufficient stiffness [3]. It has been suggested that future research should systematically alter the collar stiffnesses (CSs) in high-collar shoes while maintaining all the other shoe parameters constant to identify the unique impacts of the CS [4]. Studies comparing different shoes [5,6] have failed to isolate the effects of CS; other studies that used a specific shoe [3,7] did not control the collar conditions for subjects with different anthropometrics (e.g., ankle width). Since the protective effects of the collar require circular embracing of the ankle and shank, increasing CS might promote circumferential ankle pressure (CAP). Evidence from the literature suggests that higher CAP could lead to increased proprioception acuity and a trend toward more active ankle stiffness [8,9]. Alternatively, subjects could respond to changes in how the shoe collar felt when pressed against their ankles [4]. Thus, CAP has the potential to affect the results of studying CS; therefore, a shoe design that allows changing the CS while controlling the collar CAP during experiments is missing and needs to be addressed.

Most of the extant studies on the protective effects of high-top shoes during sports activities have been dedicated to cutting maneuvers [10] and landing [7], where the risk of lateral ankle sprain is high; however, running is also essential for athletes who wear high-collar shoes, e.g., basketball and football players. For example, on average, basketball players spend 11% of their game time in moderate-intensity running, covering approximately 3.4 km per game at a pace of 4 m/s [11]. It has been reported that lateral ankle sprain is one of the most frequent acute injuries during running, which may cause prolonged training interruptions [12]. Currently, the extent to which high-collar shoes impact running biomechanics is unknown.

The interactions of CS in regulating factors related to lateral ankle sprain during running are poorly understood, and this present study focuses on muscular activity, dynamic stability, and leg stiffness (K_leg_). The muscle activity necessary to stabilize the ankle joint appears to be influenced by CS. According to research on walking, muscle activity is likely to decrease when the demand placed on the lower limb is reduced, perhaps as a result of the superior support provided by a greater high-collar stiffness [4]; however, no studies have investigated this assumption during running. Notably, dynamic stability was used to assess the risk of ankle sprain, showing that the lack of dynamic stability could significantly increase the risk of lateral ankle sprain [13]. Enclosing the ankle and shank with a stiffer collar may provide lateral protection and improve mediolateral dynamic stability; moreover, a sufficient level of K_leg_ is essential to reduce the risk of musculoskeletal injuries during running [14]. It has been suggested that altering the leg stiffness may mitigate ankle inversion injuries [15]; however, no evidence exists on how changing the CS may affect running injury risk factors, such as dynamic stability and K_leg_. Such information may be helpful to players for preventing injuries or fostering specific training adaptations, with individuals selecting shoes associated with high or low CSs based on maximizing the advantages.

Current research seeks to address the shortcomings of previous studies by investigating the effectiveness of high-collar shoes with adjustable collar stiffness (CS) in ankle sprain prevention during running. While previous research has explored various aspects of shoe design and its impact on athletic performance, there is a lack of comprehensive studies specifically focusing on the adjustable collar stiffness feature. By utilizing a biomimetic approach, this study aims to design a shoe with improved CS adjustability and measure its impact on dynamic stability, leg stiffness, and muscular activity during running. The primary objective is to design a high-collar shoe with adjustable CS and propose a method of measuring CS. The secondary objectives are to examine the effects of CS on muscular activity, dynamic stability, and K_leg_ (kinetic leg properties) during running. It is hypothesized that higher CS will lead to higher K_leg_ and dynamic stability, as well as lower muscular activity. The findings of this study will not only enhance our understanding of the impact of high-collar shoes with adjustable CS on running mechanics but also have potential implications for other fields. For instance, previous studies have suggested that such shoe designs could improve gait stability in older individuals [16] and reduce joint pain in people with osteoarthritis [17]. Therefore, this research has the potential to benefit both athletes and individuals with specific foot-related conditions, contributing to advancements in footwear design and its practical applications.

## 2. Materials and Methods

### 2.1. Study Design

To investigate the immediate effects of a custom-designed high-collar shoe, a within-participant design was used. To investigate the effects of the custom-designed high-collar shoe on running biomechanics, an observational study design was used.

### 2.2. Setting

In this study, we recruited able-bodied young male participants in April 2022 at Chung-Ang University, South Korea. The experiments were conducted at the Assistive and Rehabilitation Robotics Laboratory of the Department of Mechanical Engineering roughly 1 week after enrollment of each participant.

### 2.3. Participants

The experimental protocol was approved by the Chung-Ang University Institutional Review Board (approval number 1041078-202106-HRZZ-165-01), and all procedures were conducted in accordance with the approved study protocol. A total of 16 participants were recruited for the study. Based on data from a previous study [5], a power calculation was conducted to determine the appropriate sample size. The calculation revealed that a minimum of 10 participants would be required to achieve 80% power and a *p* value of 0.05. To compensate for the possible subject dropout, a total of 16 participants was considered sufficient. The inclusion criteria for the participants were as follows: 27.0 cm foot length (US size: 10) and no history of pathology that could affect their running performance. Participants with any functional, neurological, or morphological disorders that could potentially impact gait were excluded from the study. All participants provided written informed consent before participation.

### 2.4. Custom-Made Footwear

A high-collar shoe can be a biomimetic design that provides support for the ankle in two ways. Firstly, the design mimics the natural ankle joint structure, which includes the bones of the lower leg and the ankle, held together by a complex network of ligaments. The high collar of the shoe mimics the ligaments’ function by providing external support to the ankle joint, reducing the risk of ankle sprains. Secondly, the high-collar shoe can mimic the natural ankle joint’s proprioceptive feedback system, which provides information about the position and movement of the joint to the brain, allowing for rapid adjustments in muscle activity to stabilize the joint. By providing external support and mimicking proprioceptive feedback, the high-collar shoe can be a biomimetic design that reduces the risk of ankle injuries during running.

**Figure 1:**
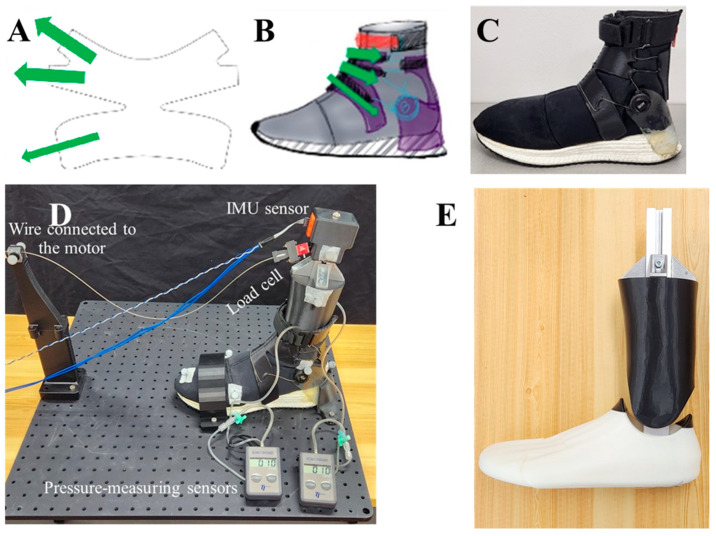
(**A**) Green arrows show the direction of the forces exerted by the top cover around the ankle; (**B**) design illustration; (**C**) manufactured prototype of the custom-designed high-collar shoe for the study; (**D**) collar stiffness measurement setup; and (**E**) foot prosthesis.

Figure 1C shows the prototype high-collar shoe that was designed and manufactured for this study (mass: 405 g; size: 10 US; length: 29 cm; collar height: 17 cm; collar diameter: 10 cm; heel height: 41 mm; heel–midfoot drop: 17 mm). To fabricate a flexible shoe suitable for running, neoprene fabric (thickness: 3 mm) was used for the upper part, and thermoplastic polyurethane (TPU) and rubber were used for the midsole (white) and outsole (black), respectively. The primary purpose of this design was to allow precise CS adjustment. This purpose was met by implementing two Boa^®^ ratcheting reels (Boa Technology Inc., Denver, CO, USA) on the lateral sides of the collar connected to zigzag-patterned wiring passed through a 3D-printed top cover made of poly lactic acid (PLA) (Figure 1B). Tightening the ratcheting reels resulted in a stiffer collar. The connecting wire and top cover were implemented so as to ensure uniform tightness around the ankle. The high-collar and heel counter are two essential components that provide external stability to the ankle [7]. This shoe prototype integrated and wired these two components to change the CS.

### 2.5. Instrumentation

#### 2.5.1. Collar Stiffness Measurement

Figure 1C shows the custom-designed high-collar shoe (size: 10 US). To fabricate a flexible shoe suitable for running, neoprene fabric (thickness: 3 mm) was used for the upper part, and thermoplastic polyurethane (TPU) and rubber were used for the midsole (white) and outsole (black), respectively. To adjust the CS, two Boa^®^ ratcheting reels (Boa Technology Inc., Denver, CO, USA) were implemented on the lateral sides of the collar connected to a zigzag-patterned wiring passed through a 3D-printed top cover made of poly lactic acid (PLA) (Figure 1A,B). The high-collar and heel counter are two essential components that provide external stability to the ankle [7]. The current shoe prototype integrated and wired these two components to change the CS.

Passive CS was measured with a 3D-printed flexion–extension foot prosthesis inserted into the shoe (Figure 1E). Figure 1D illustrates how a wire fixed to the prosthetic shaft applied the force generated by a 200 W BLDC motor (EC-4pole 30, Maxon, Sachseln, Switzerland) at a constant velocity of 10 cm/s. Starting from the 90° position, the motor pulled the shaft until it reached 60° for dorsi-flexion and 110° for plantar-flexion, measured with an IMU sensor (MTi-630, Xsens, Enschede, The Netherlands) that controlled the motor. After reaching the end range angle, the motor released the wire with the same constant speed to measure collar recoil movement. The force in the wire was measured using a Futek load cell (LSB205, Futek Inc., Irvine, CA, USA) sampled at 1000 Hz and synchronized with motion capture. The collar movement and angle of the wire pulling the prosthetic shaft were tracked using reflective markers.

At the 90° shaft position, two pressure-measuring sensors (Kikuhime, TT Meditrade, Horsens, Denmark) were placed on the medial and lateral malleolus of the prosthesis to measure the circumferential ankle pressure. Then, the Boa^®^ ratcheting reels were regulated until both sensors reached the same levels of 10-, 30-, and 60-mmHg, representing the low, medium, and high CS conditions, respectively. The pressure-measuring sensors were removed by unzipping the shoe’s back zipper.

#### 2.5.2. Motion Analysis

Kinematic information was obtained using an eight-camera T-10 Vicon motion capture system (Oxford Metrics LTD., Oxford, UK) operating at a sampling frequency of 200 Hz. A modified full-body Vicon Plug-in-Gait marker set (PiG-Vicon Motion Systems, Oxford, UK) with 74 light-reflective markers was used to generate the kinematic data and center of mass (COM) trajectory. Extra cluster markers were placed on the femur, shank, upper arm, and forearm.

#### 2.5.3. Muscular Activity

Surface electromyography (EMG) data were recorded for the gastrocnemius medialis (GM), soleus (SOL), peroneus longus (PL), tibialis anterior (TA), vastus lateralis (VL), and semitendinosus (SEMI) muscles of the dominant lower limb at 2000 Hz using the Wireless EMG System (Trigno TM Wireless System, DELSYS Inc., Natick, MA, USA). The electrodes were located following the SENIAM guidelines [18]. Initial contact and foot-off were identified using two foot-switch sensors (402, Interlink Electronics Inc., Camarillo, CA, USA) embedded in the insole of each shoe and connected to the four channels of the wireless EMG system.

### 2.6. Running Experiments

All participants ran on a treadmill with zero inclination at 4 m/s. The participants performed a combination of 5 min of walking and running for warm-up. After familiarization with the shoe and collar conditions, each participant performed three 20-s running trials with the low, medium, and high CS conditions in randomized order, with a 5-min break between trials. After setting a CS condition, slight adjustments were applied if the participants felt a difference between the right and left shoes.

### 2.7. Data Processing and Analysis

#### 2.7.1. Collar Stiffness

CS data were filtered using a fourth-order zero-lag low-pass Butterworth filter with a cut-off frequency of 10 Hz. The moment–angle curve was calculated using the component of the wire force perpendicular to the shaft of the prosthesis. The slopes at 90° to 120° and 90° to 70° characterized the dorsi-flexion and plantar-flexion CSs, respectively [5]. The area between the pulling and recoiling curves indicates the mechanical energy lost [5].

#### 2.7.2. Kinematic Data and Dynamic Stability

A total of 10 consecutive strides were selected, providing 160 trials for analyses. Reflective markers data were filtered using a fourth-order zero-phase-lag Butterworth low-pass filter with a cut-off frequency of 10 Hz. The joint ROM and COM trajectories were calculated using a 15-segment model built with Visual3D software (CMotion Inc., Rockville, MD, USA). The lateral boundary of the base of support (BOS) was estimated using the spatial location of the fifth metatarsal marker. The mediolateral dynamic stability index during the stance phase was calculated as the minimum distance between the mediolateral position of the COM transverse projection and the lateral aspect of the BOS, normalized to step width [19].

#### 2.7.3. EMG Data

EMG data were filtered with a bandpass filter (50–450 Hz). Following full-wave rectification, a fourth-order zero-phase-lag Butterworth low-pass filter with a cut-off frequency of 10 Hz was applied. The EMG data for each muscle were then normalized with the respective maximum signal across all trials of the same muscle [3]. The pre-heel-strike phase was defined as the time from 50 ms before heel contact [20]. Further, the stance phase was subdivided into the weight acceptance and propulsion phases as heel contact to maximum knee flexion and the remaining of the stance phase, respectively. Each phase was normalized to 50 points, and the EMG–time curves were integrated over the intervals to determine the muscle activity levels (IEMG).

#### 2.7.4. Leg Stiffness

The method described by Morin et al. [21] was used to calculate K_leg_, which provides the most reasonable estimate of stiffness during running compared to other mathematical models [22]. K_leg_ (kN·m^−1^) was calculated as follows:

(1) $$K_{leg}=F_{max}\times \Delta L^{-1}$$

with

(2) $$F_{max}=mg\times \left(\frac{\pi }{2}\right)\times [\left(\frac{t_{f}}{t_{s}}\right)+1]$$

and

(3) $$\Delta L=L-\sqrt{L^{2}-{\left(\frac{vt_{s}}{2}\right)}^{2}}+\Delta y$$

and:

(4) $$\Delta y=\left|-\frac{F_{max}}{m}\times \frac{t_{s}^{2}}{\pi^{2}}+g\times \frac{t_{s}^{2}}{8}\right|$$

where F_max_ is the maximal vertical force (kN); ΔL is the leg spring compression (m); t_f_ (s) is the flight time; t_s_ (s) is the stance time; m (kg) is the body mass; v (m/s) is the running velocity; and L (m) is the leg length (distance from the greater trochanter to the ground measured in an upright position). All mathematical calculations were performed using MATLAB.

### 2.8. Statistical Method

The results were expressed as mean ± standard deviation. The normality of distribution was assessed with the Shapiro–Wilk test and the normality assumption was met for all outcome measures; moreover, Levene’s test showed normal homogeneity of variance for all parameters. One-way repeated-measures analysis of variance (ANOVA) with Bonferroni’s correction was applied to determine the effects of the three collar conditions on the desired parameters. If the ANOVAs indicated significant differences, post hoc pairwise comparisons with Bonferroni adjustments were used for identification. The statistical significance was set at *p* < 0.05.

## 3. Results

**Figure 2:**
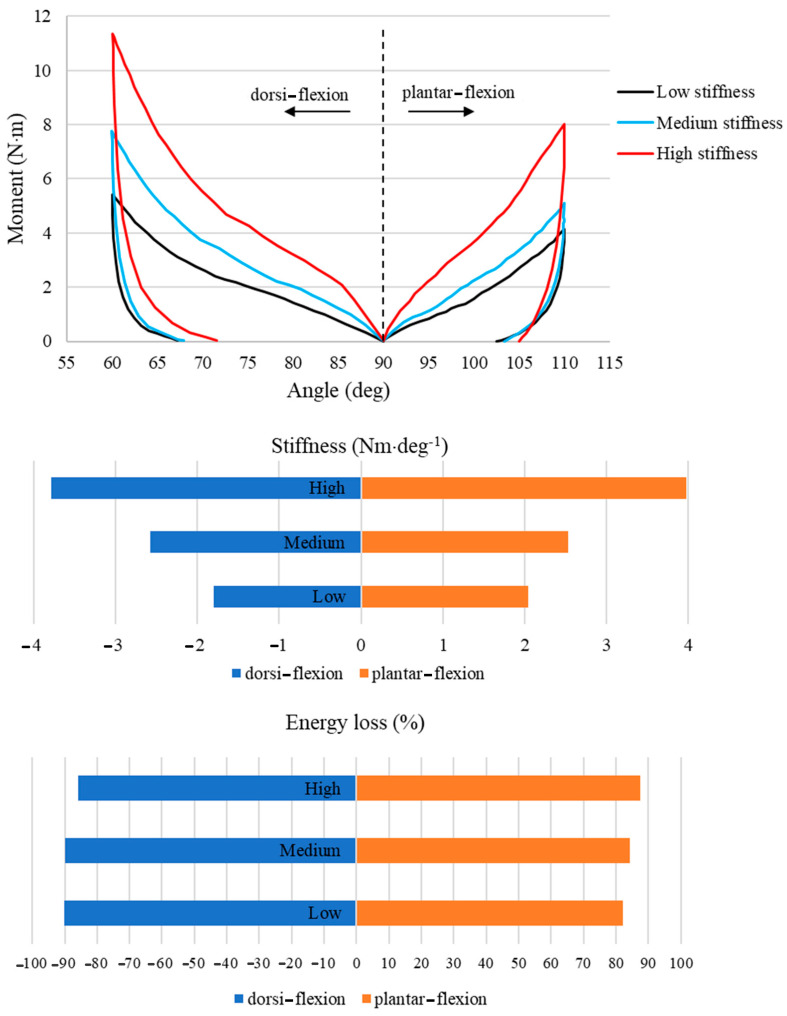
(**Top**) The moment−angle plot, (**middle**) stiffness, and (**bottom**) energy loss for three collar conditions.

Sixteen healthy active males (24.56 ± 2.56 years; 1.78 ± 0.05 m; 71.25 ± 6.12 kg) participated in this study. The stiffness characteristics assessed in the CS conditions in this study are presented in Figure 2. Compared to low stiffness, the medium and high CSs were 30% and 52% greater for the dorsi-flexion and 19% and 49% greater for plantar-flexion, respectively; moreover, the high CS was 32% and 36% greater than the medium CS condition for the dorsi-flexion and plantar-flexion, respectively. Energy losses for the dorsi-flexion and plantar-flexion were over 85% and 82%, respectively.

**Figure 3:**
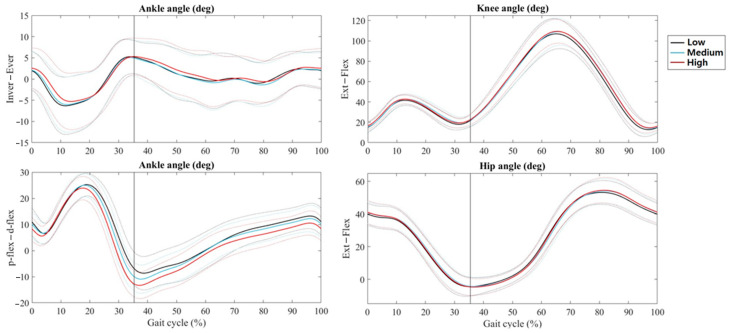
(Mean ± SD) Ankle, knee, and hip joint angles during running under the three collar conditions.

Figure 3 shows the average lower limb joint angle for the low, medium, and high CS conditions. The step frequency, width, and length did not differ among these conditions (Table 1). The ankle sagittal and frontal ROMs significantly reduced with increased CS, although the knee and hip joint ROMs did not show any changes between the conditions. Additionally, there was significantly lower dynamic stability with low stiffness compared to the other two conditions; however, the latter did not show any significant difference.

**Figure 4:**
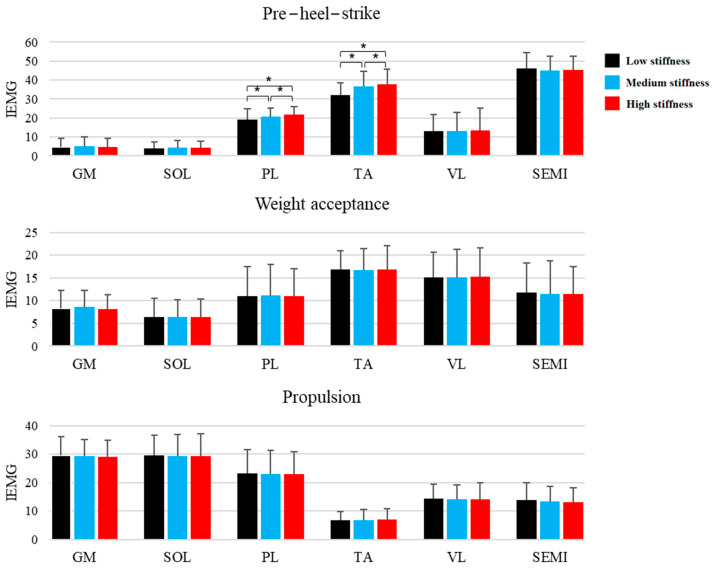
Integrated EMG (IEMG) activities of the gastrocnemius medialis (GM), soleus (SOL), peroneus longus (PL), tibialis anterior (TA), vastus lateralis (VL), and semitendinosus (SEMI) muscles during running under the three collar conditions. * *p* < 0.05.

The muscular activities of the studied muscles are represented in Figure 4. The GM, SOL, VL, and SEMI muscular activities were not different during the three phases between the shoe conditions (Figure S1). The only changes in muscular activity occurred during the pre-heel-strike phase, where the muscular activities of the PL and TA significantly increased with stiffness.

Based on the results in Table 2, t_s_ significantly decreased with an increase in stiffness, while there were no differences related to t_f_ between the conditions; further, there was a trend towards significantly more F_max_ and K_leg_ with greater CS.

## 4. Discussion

Following the study objectives, the primary purpose was to develop a high-collar shoe with adjustable collar stiffness (CS) through a biomimetic approach. As a result, mechanical testing of the high-collar shoe revealed distinctive moment–angle relations, displaying nonlinear characteristics across all CS conditions, reflecting the properties of the collar material. Furthermore, our investigation into the stiffness characteristics demonstrated that increasing collar stiffness required greater energy for deformation, particularly evident in the higher percentages of energy returned during dorsi-flexion and the corresponding lower percentages during plantar-flexion, compared to the low CS condition. These findings shed light on the complex mechanical behavior of the collar and provide valuable insights into its energy transfer properties during recoil.

Similar studies introduced different methods for measuring the collar or shaft bending stiffness. Cikajlo and Matjacic [6] assessed the load-deformation of high-collar shoes using a robot that simulated the gait stance phase. The main issue with this method is that it does not solely measure the collar stiffness but the whole shoe’s bending stiffness (including the boot shaft and the vamp). Böhm and Hösl [3] provided the CS diagram in all directions using a similar method to the current study but they only applied a range of constant forces to the shaft, and it is unclear how the high-collar reacted during different ankle angles. Kersting et al. [5] used a combination of a portable force sensor and an inclinometer to measure CS; however, the force to the collar was applied manually, questioning this method’s consistency and reliability. The main advantage of the CS measurement method presented in this study is the moment–angle plot that allows modeling the true effect of the high-collar on gait biomechanics.

This study systematically investigates shoe CS effects on muscular activity, dynamic stability, and K_leg_ during running. The results indicate that medium and high CSs reduce both sagittal and frontal ankle ROMs compared to the low collar condition. This is expected as various studies have reported the restrictive effects of a stiffer collar on the ankle joint ROM [3,5], which is the primary mechanism of preventing lateral ankle injuries. In addition, the alteration of the CS and subsequent restriction of the ankle joint movement did not produce any compensation on the knee or hip joints. This result agrees with those of previous studies [3,5], suggesting that high-collar shoes restrict ankle kinematics but not the more proximal joints.

Contrary to the second hypothesis, there were no significant differences in the muscular activities among the CS conditions during the stance phase. The demand placed on the ankle/foot complex while running was unlikely to be adequate to require changes to the lower limb muscle activity in response to alteration of the CS. However, an interesting finding was that the medium and high CS conditions caused increases in the muscular activities of the PL and TA during the pre-heel-strike phase. Theoretically, the pre-landing EMG activities of the PL and TA are considered necessary for preparing the foot for initial contact and subsequent weight acceptance [23]. Such pre-activation stiffens the muscle and restricts joint rotations after the heel strike [24]. Therefore, adequate pre-landing muscular activity is considered a protective mechanism for the ankle joint and ligaments at touchdown [23]; however, it is difficult to explain what intrinsic mechanisms caused the differences between the CS conditions because the effects of high-collar shoes on ankle evertor muscle function have not been investigated systematically during running. It has been shown that adding compression around the ankle may improve ankle proprioception [8,25]. Since the investigated high-collar shoe completely covered the ankle and the change in CS was accompanied by changing CAP, proprioception may be improved during running. It has been suggested that the elevated tactile sensory and proprioceptive inputs of the ankle joint, as a result of the higher CAP, may influence the magnitude of pre-heel-strike PL and TA muscle activities [23], which could explain the differences in EMG activities among the CS conditions. Further, our findings contrast with another study [23] that identified decreased pre-landing evertor muscle activity while wearing a high-collar shoe during an unexpected landing on a tilted surface. Besides the differences between the high-collar shoes and investigated movements, researchers did not consider CS, making it difficult to compare their results with those of this present study.

Notably, a lack of postural stability might significantly increase the risk of lateral ankle sprains [13]. Our findings indicate that medium and high CSs may lead to greater dynamic stability than low CS during running. This finding confirms the hypothesis that higher CS improves dynamic stability during running. No concurrent change in the step width suggests that higher stiffness restricts the mediolateral excursion of COM rather than adjusting foot placement (COM mediolateral excursions were 0.028 ± 0.007, 0.020 ± 0.006, and 0.019 ± 0.006 for the low, medium, and high CSs, respectively; *p* < 0.001). According to Perry et al. [19], changes in the footwear characteristics impact mechanical support and the interface between the foot and environment, which could constrain the range of COM movements over the BOS. In our experiments, the only change in shoe properties was the CS; it seems that with higher CS, more mechanical support may be offered to counteract the excursion of COM; in addition, higher CS was correlated with higher CAP, which may improve the ankle tactile sensory inputs. There is strong evidence that applying CAP may lead to better postural stability by improving the ankle’s active stiffness and proprioceptive acuity [8]. Proprioceptive information provided by greater high-collar stiffness can assist individuals in maintaining stability by helping the maintenance of their COM well within the limits of the BOS [26]. Therefore, it appears that enclosing the ankle and shank with a stiffer high collar may restrict COM mediolateral excursion and improve dynamic stability by providing mechanical support and/or improved proprioception owing to greater CAP.

Based on the present findings, there was a trend toward higher K_leg_ with increased CS. This result, caused by the reduced contact time experimented with higher CS, partially confirms the second hypothesis. In this study, a higher CS was equivalent to a higher CAP, meaning more ankle tactile sensory inputs. Evidence supports the idea of a higher K_leg_ following improved ankle tactile sensory inputs. In fact, improved tactile inputs while wearing shoes may modulate muscle activation and/or joint kinematics to enhance stiffness and potentiate the stretch–shortening cycle to adjust running performance to the new condition [27]. Although it has been suggested that changing K_leg_ may increase the risk of inversion injuries [15], and a higher K_leg_ has been found in individuals who are more prone to lateral ankle sprains [28], the relationship between K_leg_ and lateral ankle sprain remained unclear and inconclusive. Nevertheless, high K_leg_ has been linked to increased loading rate and bone stress [29]; therefore, athletes susceptible to bone-related injuries may consider stiffer high-collar shoes with cushioning. However, the appropriate amount of K_leg_ for high-collar shoes during running is likely to vary depending on the running technique, anthropometry, and physical capabilities, and is yet to be determined; therefore, future investigations may need to provide more support for this assumption.

The findings of this study have important implications for research in the field of ankle sprain prevention and footwear design. By systematically investigating the effects of collar stiffness (CS) on muscular activity, dynamic stability, and leg stiffness during running, we have provided valuable insights into the biomechanical responses associated with high-collar shoes. Our study demonstrates that higher CS conditions significantly reduce ankle range of motion and improve dynamic stability, indicating the potential of high-collar shoes to enhance ankle joint support and reduce the risk of lateral ankle sprains. Moreover, the trend towards higher leg stiffness with increased CS suggests that high-collar shoes may facilitate better energy transfer and running performance. These findings contribute to the growing body of knowledge on designing and optimizing high-collar shoes and provide a basis for further research in developing innovative footwear technologies for injury prevention and performance enhancement in sports and high-demand activities.

## 5. Limitations

There are limitations in our study that need to be considered. First of all, the lack of confounding testing, which should be acknowledged. Confounding testing refers to the examination of potential variables or factors that could influence the outcomes of our study but were not specifically addressed or controlled for. By not incorporating confounding testing, we may have overlooked certain variables that could have influenced the observed results and introduced potential bias. Furthermore, we did not quantify the collar inversion–eversion stiffness due to the constraints of the prosthetic foot movement. This omission limits our understanding of the complete biomechanical implications of the collar design. Future research should consider this to provide a more comprehensive analysis. Additionally, in some participants, we had to place the medial or lateral malleolus ankle markers directly on the ratcheting reels. To compensate for this placement, we created a virtual marker in Visual3D by subtracting the height of the ratcheting reel. Although we took measures to mitigate potential errors, this approach introduces a potential source of measurement imprecision that could have affected the accuracy of our results. Finally, it is crucial to interpret our findings with caution when extrapolating them to real-life situations, as our results only pertain to the specific shoe design investigated in this study. Different shoe designs, materials, and individual variations may yield different outcomes; therefore, the applicability of our findings to broader contexts should be examined in future studies encompassing a wider range of shoe designs and diverse populations. Overall, while our study provides valuable insights into the biomechanical aspects of the collar design and its implications for running mechanics, it is important to acknowledge these limitations and consider them when interpreting and applying our findings.

## 6. Conclusions

In conclusion, our study highlights the importance of a biomimetic design approach in developing high-collar shoes to provide stability for the ankle joint. Based on our findings, a greater CS has a limiting effect on the ankle joint ROM but not on those of the knee and hip joints. Although CS appears to play a role in regulating the muscle activation required to stabilize the ankle joint during the pre-heel-strike phase, it did not affect the muscular activity during the stance phase; moreover, the restrictive nature of higher CS improved dynamic stability, and there was a trend toward higher K_leg_ with increased CS. Thus, higher CS may improve some parameters, leading to better support against lateral ankle sprain during running. It was observed that collar CAP might play a significant role in regulating collar properties, so we suggest considering CAP when studying CS. However, it is essential to note that the conclusions of this study should be interpreted with caution due to the study design and the absence of ankle sprain measurements. Therefore, further research involving larger-scale, higher-quality studies is warranted to confirm and strengthen these findings. The findings of this study may be considered when designing or purchasing high-collar shoes.

## Figures and Tables

**Table 1:** (Mean ± SD) Spatiotemporal parameters, joint angles, and dynamic stability index for running under three collar conditions.

Stiffness	Low	Medium	High	*p*-Value
Step frequency (Hz)	180.07 ± 4.51	181.84 ± 4.55	182.89 ± 4.81	0.617
Step width (cm)	6.50 ± 3.22	6.44 ± 2.99	6.31 ± 3.03	0.541
Step length (cm)	134.95 ± 11.37	135.12 ± 10.09	134.92 ± 9.16	0.873
Ankle ROM—sagittal (deg)	35.28 ± 3.82 ^a,b^	34.98 ± 4.45 ^c^	34.25 ± 5.20	0.001 *
Ankle ROM—frontal (deg)	11.57 ± 4.51 ^a,b^	11.09 ± 4.57 ^c^	10.55 ± 4.31	0.001 *
Knee ROM (deg)	94.25 ± 11.81	95.18 ± 14.25	95.27 ± 15.11	0.115
Hip ROM (deg)	58.82 ± 6.17 ^a,b^	58.64 ± 6.35 ^c^	58.60 ± 6.55	0.355
Dynamic stability index	1.65 ± 0.79 ^a,b^	1.78 ± 0.65	1.80 ± 0.72	0.002 *

^a^ Significant differences between low and medium stiffness conditions. ^b^ Significant differences between low and high stiffness conditions. ^c^ Significant differences between medium and high stiffness conditions. * *p* < 0.05.

**Table 2:** (Mean ± SD) K_leg_ measures for running under three collar conditions.

Stiffness	Low	Medium	High	*p*-Value
t_s_ (s)	0.251 ± 0.015 ^a,b^	0.247 ± 0.015 ^c^	0.243 ± 0.013	0.001 *
t_f_ (s)	0.440 ± 0.042	0.441 ± 0.035	0.443 ± 0.035	0.315
F_max_ (kN)	2.997 ± 0.342 ^a,b^	3.030 ± 0.297 ^c^	3.071 ± 0.304	0.001 *
K_leg_ (kN·m^−1^)	13.681 ± 3.282 ^a,b^	14.235 ± 3.075 ^c^	14.852 ± 2.589	0.001 *

^a^ Significant differences between low and medium stiffness conditions. ^b^ Significant differences between low and high stiffness conditions. ^c^ Significant differences between medium and high stiffness conditions. * *p* < 0.05.

## Data Availability

Data supporting the findings of this study are available upon request from the first author.

## Supplementary Materials

The following supporting information can be downloaded at: https://www.mdpi.com/article/10.3390/biomimetics8030274/s1, Table S1. Muscular activities (mean ± SD) for gastrocnemius medialis (GM), soleus (SOL), peroneus longus (PL), tibialis anterior (TA), vastus lateralis (VL), and semitendinosus (SEMI) muscles during running under three shoe conditions.
