# Supplementary material for: The Effects of a Custom−Designed High−Collar Shoe on Muscular Activity, Dynamic Stability, and Leg Stiffness: A Biomimetic Approach Study
Source: Biomimetics (Basel). 2023 Jun 27;8(3):274. doi: 10.3390/biomimetics8030274 (PMC10377164; doi:10.3390/biomimetics8030274)
Supplement: Supplementary file 1 [file biomimetics-08-00274-s001.zip › biomimetics-2407782-supplementary.pdf]

Table S1. Muscular activities (mean ± SD) for gastrocnemius medialis (GM), soleus (SOL), peroneus longus (PL), tibialis anterior (TA), vastus lateralis (VL), and semitendinosus (SEMI) muscles during running under three shoe conditions.

	**Pre–heel–strike**				**Weight acceptance**				**Propulsion**			
**Stiffness**	Low	Medium	High	p-value	Low	Medium	High	p-value	Low	Medium	High	p-value
GM	4.78 ± 4.35	4.95 ± 4.93	4.67 ± 4.58	0.40	8.23 ± 3.96	8.36 ± 3.57	8.16 ± 3.13	0.08	29.67 ± 6.53	29.37 ± 5.69	29.05 ± 5.90	0.07
SOL	4.06 ± 3.29	4.31 ± 3.70	4.22 ± 3.69	0.54	6.42 ± 4.05	6.39 ± 3.77	6.48 ± 3.92	0.43	29.57 ± 7.05	29.34 ± 7.55	29.24 ± 7.83	0.37
PL	19.32 ± 19.30 [a,b]	20.58 ± 19.51 [c]	21.73 ± 22.52	0.01 *	11.06 ± 6.39	11.19 ± 6.71	11.05 ± 5.94	0.32	23.19 ± 8.47	22.90 ± 8.40	23.02 ± 7.87	0.78
TA	32.13 ± 26.51 [a,b]	36.59 ± 27.84 [c]	37.72 ± 28.92	0.01 *	16.82 ± 4.21	16.62 ± 4.76	16.96 ± 5.18	0.11	6.76 ± 3.12	6.88 ± 3.59	6.91 ± 3.82	0.21
VL	13.05 ± 8.78	13.24 ± 9.75	13.51 ± 11.69	0.06	15.15 ± 5.53	15.18 ± 6.18	15.29 ± 6.38	0.17	14.46 ± 5.03	14.08 ± 5.21	14.16 ± 5.66	0.31
SEMI	46.25 ± 28.19	44.89 ± 27.65	45.26 ± 27.49	0.08	11.78 ± 6.47	11.49 ± 7.31	11.44 ± 6.11	0.28	13.91 ± 6.07	13.41 ± 5.36	13.31 ± 5.12	0.07

a Significant differences between low and medium stiffness conditions.
b Significant differences between low and high stiffness conditions.
c Significant differences between medium and high stiffness conditions.
* $p < 0.05$
